# Time-series transcriptome and metabolome profiling reveals *GhWRKY33* as positive regulator in cotton response to *Verticillium dahliae* infection

**DOI:** 10.3389/fpls.2026.1959387

**Published:** 2026-10-28

**Authors:** Wanli Han, Gang Wang, Zhibin Zhang, Fuxiang Zhao, Xuwen Wang, Tao Yang, Shidong Wang, Li Liu, Jinxin Qiao, Aijun Si, Yu Yu, Xianhui Kong

**Affiliations:** 1 Cotton Research Institute, Xinjiang Science Academy of Agriculture and Reclamation, Ministry of Agriculture, Shihezi, Xinjiang, China; 2 Cotton Research Institute, Chinese Academy of Agricultural Sciences, Anyang, China; 3 College of Modern Agriculture and Food Engineering, Xinjiang Vocational University of Technology, Kashi, Xinjiang, China

**Keywords:** *GhWRKY33*, *Gossypium hirsutum L.*, metabolomic, transcriptomic, *Verticillium dahliae*

## Abstract

Verticillium wilt severely limits global cotton production, yet the molecular basis of early resistance in upland cotton remains poorly understood. Here, we integrated time-series transcriptomics and untargeted metabolomics to resolve early defense dynamics in cotton seedling roots of the VW-resistant cultivar Jinken1775 and the susceptible cultivar Junmian1 at 0, 4, 8, 12, 24, and 72 h post-inoculation (hpi) with *Verticillium dahliae*. Results revealed distinct early defense dynamics. The resistant genotype exhibited restrained yet sustained transcriptional activation coupled with preferential flavonoid/lipid accumulation, whereas the susceptible cultivar displayed disordered reprogramming. Integrated analysis of DEGs and DAMs resolved resistance circuits enriched in calcium signaling, membrane remodeling, and secondary metabolism. Network inference positioned *GhWRKY33* as a central hub connecting early signal transduction with downstream metabolic reprogramming. Virus-induced gene silencing of *GhWRKY33* significantly compromised cotton resistance to *V. dahliae*, indicating its positive regulatory role in the defense response. Haplotype analysis across 419 upland cotton accessions further revealed that favorable *GhWRKY33* alleles are preferentially enriched in resistant lines. This study provided molecular targets for genetic improvement of disease resistance.

## 1 Introduction

Cotton (*Gossypium hirsutum* L.) is a globally important economic crop, yet production is severely threatened by Verticillium wilt (VW), a devastating vascular disease caused by the soil-borne fungus *Verticillium dahliae* (*V. dahliae*) (Ma et al., 2021; Wang et al., 2022; Zhang et al., 2022; Zhu et al., 2023; Adeleke, 2024). Owing to its intractable nature, VW has long been referred to as the “cancer of cotton” (Cheng et al., 2025). To date, only a limited number of resistant germplasms and resistance genes have been identified (Klosterman et al., 2009). Moreover, the continuous host–pathogen arms race between *V. dahliae* and cotton accelerates pathogen evolution, often resulting in the rapid breakdown of resistance in newly released cultivars (Zhang et al., 2025). Therefore, discovering new disease-resistant genes and breeding durable resistant varieties are essential for effective VW management (Li et al., 2021), and they are also the most cost-effective and environmentally friendly strategies for disease management (Tinggang et al., 2022).

Plants have evolved multilayered defense against *V. dahliae* infection, including activation of innate immunity, reinforcement of cell walls, and reprogramming of secondary metabolism (Nürnberger et al., 2004; Zhou and Zhang, 2020; Ngou et al., 2022). Innate immunity constitutes the first inducible barrier and comprises pattern-triggered immunity (PTI) and effector-triggered immunity (ETI) (Li et al., 2016). PTI is initiated by pattern recognition receptors (PRRs) that perceive pathogen- or damage-associated molecular patterns (PAMPs/MAMPs and DAMPs), thereby activating early defense responses such as Ca²^+^ influx and secondary metabolite biosynthesis (Lecourieux et al., 2006; Macho and Zipfel, 2014; Kim et al., 2017; Alves et al., 2021; Zhu et al., 2025). Calcium-dependent protein kinases (CDPKs) act as primary Ca²^+^ sensors that decode calcium signatures to regulate transcriptional reprogramming and hormone signaling (Matschi et al., 2015; Li et al., 2016). *Arabidopsis* CPK28 and rice CPK4/18 negatively regulate plant immune signaling, and cotton CDPK28 negatively regulate VW resistance (Monaghan, 2018; Wang et al., 2018; Wu et al., 2021b). Beyond signaling, the cell wall provides a crucial physical barrier against pathogen invasion, with lignin deposition representing a hallmark of defense (Tang et al., 2019; Narváez-Barragán et al., 2022). Lignin biosynthesis, derived from the phenylpropanoid pathway, strengthens the wall through monolignol polymerization, thereby restricting pathogen entry and spread (Bonello and Blodgett, 2003; La Camera et al., 2004; Zhao et al., 2026). In cotton, enhanced lignification limits *V. dahliae* hyphal expansion and suppresses toxin secretion and cell wall-degrading enzymes, ultimately constraining pathogen growth (Ma, 2024). Resistant cultivars generally exhibit higher lignin content and elevated expression of lignin biosynthetic genes (Xu et al., 2011; Yang et al., 2021), including *GhLAC*, *GhCAD*, *Gh4CL*, and *GhF5H* (Zhang et al., 2019; Li et al., 2022; Alariqi et al., 2023), all of which have been functionally linked to VW resistance. In parallel with structural defenses, signaling and transcriptional regulators play central roles in orchestrating immune responses. The jasmonic acid (JA)-associated regulator *GhOPR9* and its interacting partner *GhRFS6* positively modulate resistance, and overexpression of *GhRFS6* enhances tolerance to *V. dahliae* (Zhu et al., 2021; Chang et al., 2023). Pathogenesis-related (PR) genes serve as classical markers of immune activation (Du et al., 2017), while WRKY transcription factors (e.g., *GhWRKY53* (Li et al., 2023), *GhWRKY55* (Ma et al., 2024), *GhWRKY41* (Xiao et al., 2023), and *GhWRKY70D13* (Xiong et al., 2020)) and LRR-RLK receptors (Sun et al., 2018) modulate cotton immunity through salicylic acid (SA), jasmonic acid (JA), and ethylene (ET) signaling pathways and downstream defense execution. In *Arabidopsis thaliana*, *WRKY33* is required for resistance to *Botrytis cinerea* and *Alternaria brassicicola* (Zheng et al., 2006) and coordinates hormonal and metabolic responses during *B. cinerea* infection, including salicylic acid- and jasmonic acid-associated defense responses (Birkenbihl et al., 2012). Together, these findings provide a biological rationale for investigating *GhWRKY33* as a candidate regulator of cotton resistance to *V. dahliae*.

Although integrated transcriptomic and metabolomic approaches have been widely adopted in plant research, their application to dissecting VW resistance in cotton seedlings has remained limited. In this study, we combined time-course transcriptomics and untargeted metabolomics to profile root responses in the VW-resistant upland cotton cultivar Jinken1775 and the susceptible cultivar Junmian1 at 0, 4, 8, 12, 24, and 72 hours post-inoculation with *V. dahliae*. This high-resolution analysis revealed persistently activated resistance-associated transcriptional modules together with preferential and sustained engagement of lipid- and flavonoid-derived metabolic pathways in the resistant genotype. Functional validation confirmed *GhWRKY33* as a central positive regulator linking early calcium signaling to downstream metabolic reprogramming, making it and its associated pathways high-priority targets for breeding durable VW resistance in cotton.

## 2 Materials and methods

### 2.1 Plant material and growth conditions

Seeds of Jinken1775 and Junmian1 were obtained from the Xinjiang Academy of Agricultural Reclamation Sciences (Shihezi, China). Seeds were surface-sterilized with 3% sodium hypochlorite for 5 min, germinated on moist filter paper at 28 °C for 48 h, and transplanted into pots containing sterilized vermiculite:perlite (3:1, v/v). Seedlings were grown in a controlled chamber under a 14 h light/10 h dark photoperiod at 25/22 °C (day/night) and 70% relative humidity until the three-leaf stage.

### 2.2 *Verticillium dahliae* infection and sample collection

The *V. dahliae* isolate V991 was cultured on potato dextrose agar at 25 °C for 5 days. Conidia were collected, filtered, and adjusted to 1 × 10^6^ spores mL^-^¹ in sterile water containing 0.02% Tween-20. At the three-leaf stage, seedlings were inoculated using a root-cutting dip method, in which trimmed roots were immersed in the conidial suspension. Mock controls were treated with Tween-20 solution only. Root tissues were harvested at 0, 4, 8, 12, 24, and 72 h post-inoculation (hpi), immediately frozen in liquid nitrogen, and stored at −80 °C. Each treatment included three biological replicates, each consisting of at least five seedlings. Superoxide dismutase (SOD) and peroxidase (POD) activities were measured using commercial kits (Comin Biotechnology, Suzhou, China). Statistical significance was evaluated using Student’s *t*-test.

### 2.3 RNA sequencing and differential expression analysis

Total RNA of root tissues was extracted using TRIzol reagent (Invitrogen, USA) and treated with DNase I (Qiagen, Germany). RNA integrity was assessed using an Agilent 2100 Bioanalyzer, and samples with RIN ≥ 8.0 were used for library construction. Strand-specific libraries were prepared with the NEBNext Ultra II RNA Library Prep Kit and sequenced on an Illumina NovaSeq 6000 platform (PE150). Raw reads were filtered with Trimmomatic v0.39 (Bolger et al., 2014) and aligned to the *G. hirsutum* TM-1 reference genome (v2.1) using HISAT (Kim et al., 2015). Gene-level raw counts was quantified with featureCounts (Liao et al., 2014), whereas FPKM values were calculated for visualization and expression-profile presentation. DEGs were identified using DESeq2 (Love et al., 2014) with thresholds of |log_2_FC| ≥ 1 and FDR < 0.05. K-means clustering was performed using ClusterGvis (https://github.com/junjunlab/ClusterGVis), and GO and KEGG enrichment analyses were conducted with clusterProfiler (Wu et al., 2021a).

### 2.4 LC–MS metabolite profiling and DAM identification

Root samples (50 mg) were ground in liquid nitrogen and extracted with 1, 000 μL methanol:acetonitrile:water (2:2:1, v/v/v) containing a mixture of isotope-labeled internal standards. After homogenization and sonication in an ice-water bath, the extracts were incubated at −40 °C for 1 h and centrifuged at 13, 800 × g for 15 min at 4 °C. The supernatants were transferred to autosampler vials for LC–MS analysis. A pooled quality-control (QC) sample was prepared by combining equal aliquots of all samples and analyzed throughout the analytical sequence to monitor system stability. Untargeted metabolomics was performed on a Vanquish ultra-high-performance liquid chromatography system (Thermo Fisher Scientific) coupled to an Orbitrap Exploris 120 high-resolution mass spectrometer (Thermo Fisher Scientific), equipped with a Phenomenex Kinetex C18 column (2.1 × 50 mm, 2.6 μm). The mobile phases were 0.01% acetic acid in water (A) and isopropanol:acetonitrile (1:1, v/v; B), with a gradient elution. The injection volume was 2 μL, and the autosampler was maintained at 4 °C. Data were acquired in both positive- and negative-ion electrospray modes at a full-MS resolution of 60, 000 and an MS/MS resolution of 15, 000, with stepped normalized collision energies of 20, 30, and 40. Raw LC–MS data were converted to mzXML format using ProteoWizard and processed using an in-house R workflow incorporating XCMS for peak detection, extraction, alignment, and integration. Metabolite annotation was performed by matching retention time, precursor-ion mass-to-charge ratio (MS1), and tandem mass spectra (MS/MS) against an in-house reference library and public spectral databases. Annotation confidence was classified following the MSI criteria. Level 1 annotations require matching retention time, accurate mass, and MS/MS spectra with authentic standards analyzed under identical LC–MS conditions. Level 2 annotations were assigned to metabolites with high-score experimental MS/MS spectra matched against reference spectral libraries, with a single plausible structural candidate. Lower-confidence features were distinguished from Level 1 and Level 2 annotations and were not considered equivalent to confirmed metabolite identities. PCA and OPLS-DA were performed using R and MetaboAnalystR. Metabolites with variable importance in projection (VIP) ≥ 1, *p* < 0.05, and |log_2_ (fold change) | ≥ 1 were defined as DAMs.

### 2.5 Co-expression network and hub gene identification

KEGG enrichment of DEGs and DAMs was conducted using clusterProfiler v4 (Wu et al., 2021a). Weighted gene co-expression network analysis (WGCNA) (Langfelder and Horvath, 2008) was conducted using the Candidate DEGs and DAMs related to resistance to VW. A soft threshold power of eight was applied to construct a scale-free network. Module–trait associations were calculated using metabolites content as external traits. Pearson correlation analysis was used to calculate gene–metabolite associations, and significant pairs (*r* > 0.85, FDR < 0.01) were visualized with Cytoscape v3.9.1 (Shannon et al., 2003).

### 2.6 Quantitative real-time PCR validation

Total RNA was isolated using the RNAprep Pure Kit (Tiangen, China), and cDNA was synthesized using the M-MLV RTase Kit (TaKaRa, Japan). qRT-PCR was performed on a Bio-Rad CFX96 system with iTaq Universal SYBR Green Supermix, using GhUBQ7 as the reference gene. Relative expression levels were calculated using the 2^–ΔCt^ method.

### 2.7 Virus induced gene silencing

TRV-mediated virus-induced gene silencing (VIGS) was performed in Jinken1775 using a previously described cotton VIGS procedure with modifications (Gao et al., 2011). The 257-bp fragment of *GhWRKY33* was selected. BLAST searches against the *G. hirsutum* TM-1 reference genome (v2.1) were performed to verify sequence uniqueness, confirm silencing specificity and rule out potential off-target silencing of closely related group IIa/IIb *WRKY* genes. This fragment was cloned into pTRV2. The recombinant pTRV2 construct and pTRV1 were separately transformed into *Agrobacterium tumefaciens* strain GV3101. Bacterial cultures were adjusted to an OD_600_ of 0.8 and infiltrated into cotyledons of one-week-old Jinken1775 seedlings. Silencing efficiency was determined by qRT-PCR at the three-leaf stage. Plants with confirmed gene silencing were then inoculated with *V. dahliae* for disease resistance assay, with TRV:00 serving as the empty-vector control. At 5 dpi, stems from *TRV: GhWRKY33* and *TRV:00* plants were longitudinally split and photographed to assess vascular browning. For fungal recovery assays, stem segments were surface-sterilized with 70% ethanol for 30s followed by 3% (v/v) sodium hypochlorite for 3min, rinsed three times with sterile water, placed on potato dextrose agar (PDA) supplemented with 50 mg/L cefotaxime, and incubated at 25 °C for 5 days.

### 2.8 Haplotype analysis for candidate gene

Haplotype analysis was conducted based on high-quality biallelic SNPs located in *GhWRKY33* and its flanking regions (± 2 kb). Prior to analysis, sites with a minor allele frequency (MAF) below 0.05, missing data, or multiallelic variants were removed, and accessions with more than 5% missing genotypes were excluded. Only homozygous SNPs were retained for haplotype inference. For each accession, alleles at all retained SNP sites were concatenated in genomic order to form haplotype sequences. Unique haplotypes were identified using the R package pegas. Haplotype frequencies were calculated, with rare haplotypes subsequently discarded and the relationships among the remaining haplotypes visualized via a median-joining network in PopART.

## 3 Results

### 3.1 Phenotypic and physiological response of cotton to *V. dahliae* infection

To evaluate cotton responses to *V. dahliae* infection, Disease phenotypes were compared between the resistant cultivar Jinken1775 and the susceptible cultivar Junmian1 following inoculation with *V. dahliae* V991. Uninoculated plants of both cultivars were included as controls (**Figure 1A**, upper panels). At 5 days post-inoculation (dpi), inoculated Junmian1 plants exhibited more pronounced leaf yellowing and wilting than Jinken1775 plants (**Figure 1A**, lower panels). Consistent with these phenotypic differences, the disease index was significantly higher in Junmian1, reaching approximately 40, compared with approximately 11 in Jinken1775 (**Figure 1B**), confirming the markedly higher resistance of Jinken1775. SOD and POD activities were subsequently examined to characterize the physiological differences between the cultivars (**Figures 1C, D**). In Jinken1775, SOD activity increased at 4 h after inoculation, declined at 8 h, and increased again at 12 h, remaining relatively stable through 72 h. SOD activity was significantly higher in Jinken1775 than in Junmian1 at 4, 12, 24, and 72 h (**Figure 1C**). POD activity showed an overall declining trend in both cultivars, with Jinken1775 maintaining significantly higher activity at each sampled time point and in the CK samples (**Figure 1D**). Together, the disease phenotypes and enzyme activity profiles distinguish the two cultivars and support their use for investigating molecular processes associated with contrasting VW resistance.

**Figure 1:**
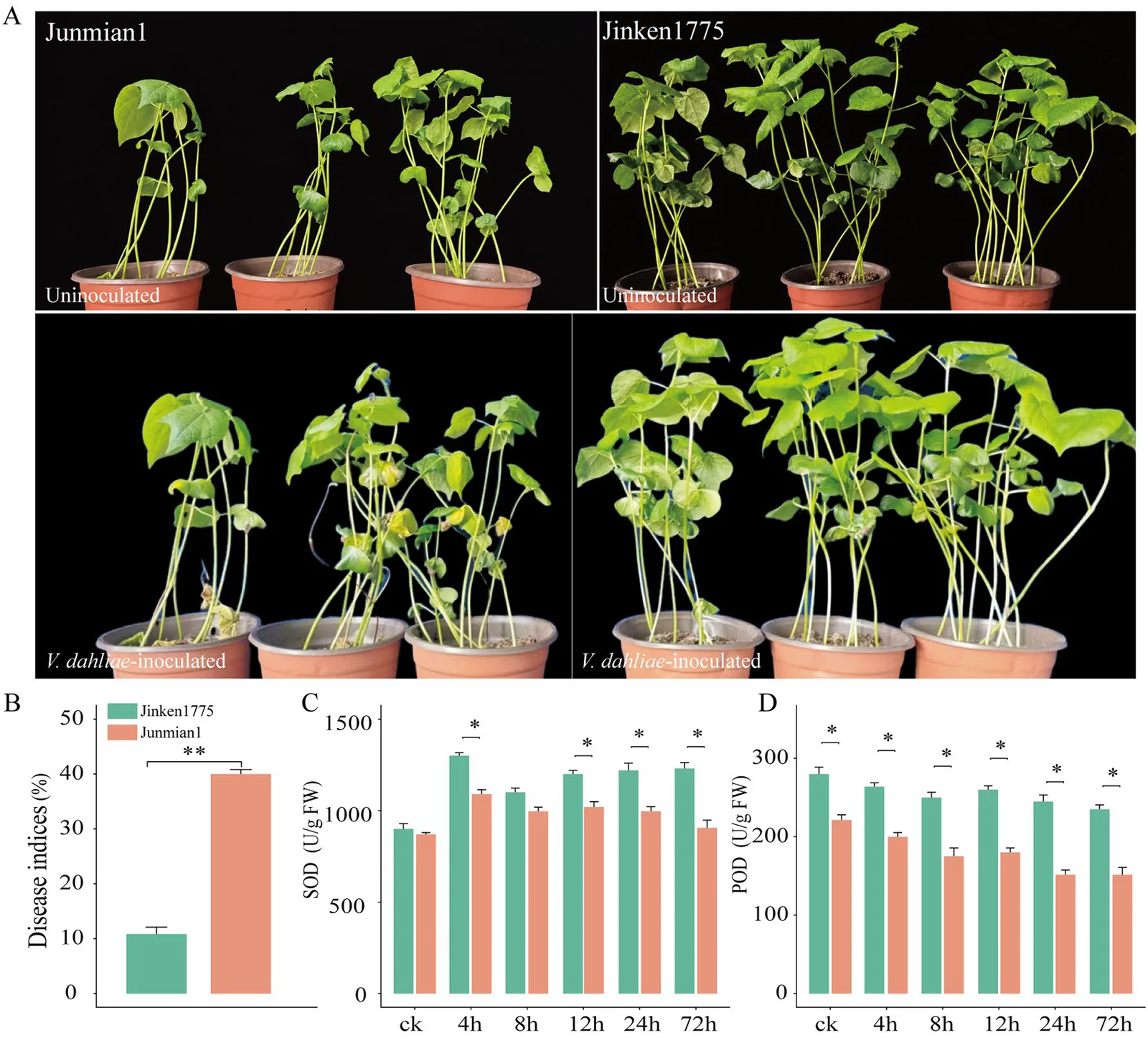
Phenotypic and physiological characteristics of upland cotton after *V. dahliae* infection. **(A)** Representative phenotypes of Junmian1 (left) and Jinken1775 (right). Upper panels show uninoculated control plants, and lower panels show plants at 5 days after inoculation with *V. dahliae* V991. **(B)** The disease index of Jinken1775 and Junmian1. **(C, D)** Temporal dynamics of superoxide dismutase (SOD) and peroxidase (POD) activities in leaves at 0, 4, 8, 12, 24, and 72 hpi are mean ± SE from three biological replicates (*P < 0.05, Student’s *t*-test). The double‑asterisk symbol (**) in **Figure 1** denotes statistically significant difference at P < 0.01 based on Student’s t‑test.

### 3.2 Time-course transcriptome profiling of Jinken1775 and Junmian1 during *V. dahliae* infection

To elucidate the dynamic transcriptional responses to *V. dahliae*, we conducted time-course RNA-seq profiling of root tissues from Jinken1775 and Junmian1. 250.98 Gb of clean data were obtained, with high sequencing quality (Q30 > 92.66%) and an average mapping rate of ~93.5% to the *G. hirsutum* TM-1 reference genome (**Supplementary Table S1**). PCA clearly separated samples by treatment and genotype, with PC1 and PC2 accounting for 23.63% and 11.20% of the variance, respectively (**Figure 2A**), indicating robust data quality and pronounced transcriptomic divergence between cultivars following infection. A total of 13, 597 unique DEGs were identified across the six sampling time points (0, 4, 8, 12, 24, and 72 hpi) between Jinken1775 and Junmian1, with each gene counted once. Comparative analysis revealed pronounced differences in transcriptional amplitude: the resistant Jinken1775 exhibited a relatively restrained response, peaking at 3, 226 DEGs at any single time point, whereas the susceptible Junmian1 showed extensive transcriptional perturbation, reaching a maximum of 8, 946 DEGs. Pairwise inter-cultivar comparisons identified 3, 658, 5, 966, 3, 104, 7, 012, 7, 471, and 8, 051 DEGs at 0, 4, 8, 12, 24, and 72 hpi, respectively, of which 1, 437, 2, 283, 1, 756, 2, 773, 3, 267, and 4, 561 were up-regulated (**Figure 2B**). UpSet analysis further uncovered 356 consistently up-regulated (**Supplementary Figure S1A**) and 263 consistently down-regulated genes (**Supplementary Figure S1B**) shared across all time points, representing a core VW-responsive gene set.

**Figure 2:**
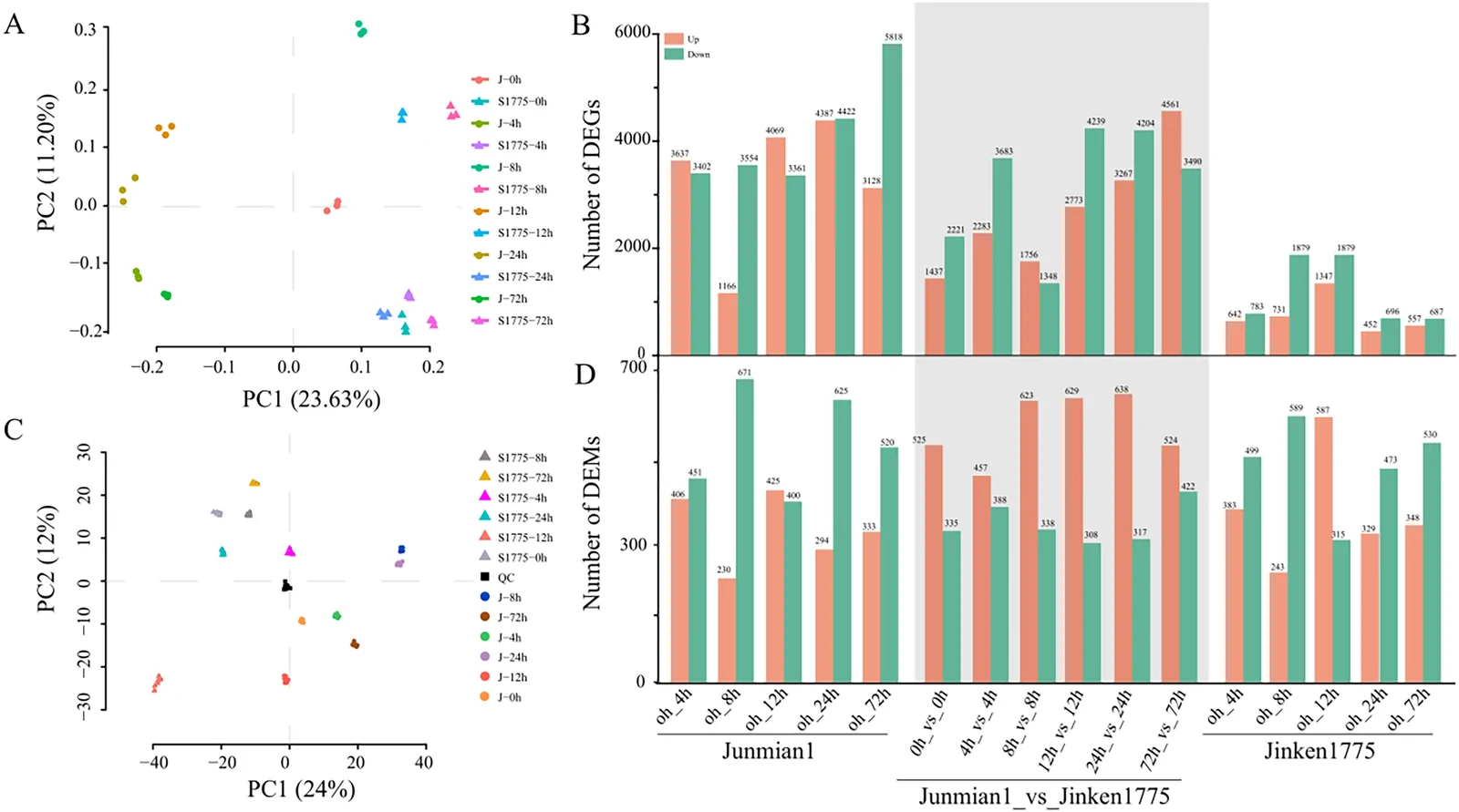
Differentially expressed genes (DEGs) analysis and differentially accumulated metabolites (DAMs) at different time points during *V. dahliae* infection. **(A)** Principal component analysis (PCA) of gene expression profiles from different samples of Jinken1775 (S1775) and Junmian1 (J) at 0, 4, 8, 12, 24, and 72 hpi. **(B)** Numbers of DEGs between Jinken1775 and Junmian1 at each time point. **(C)** PCA of metabolomic profiles of Jinken1775 and Junmian1. **(D)** Numbers of DAMs between Jinken1775 and Junmian1.

To further resolve resistance-associated transcriptional programs, all DEGs were subjected to K-means clustering based on temporal expression patterns. Ten clusters (C1-C10) were identified (**Figure 3**). Among them, cluster C8 displayed a sustained and preferential induction in Jinken1775 across middle and late infection stages, whereas its activation in Junmian1 was markedly weaker. This genotype-biased pattern suggests that C8 represents a resistance-associated transcriptional module. In contrast, other clusters showed either transient or non-specific responses. Cell cycle-related clusters (e.g., C4 and C10) were briefly induced at early stages in both cultivars, photosynthesis-related clusters (e.g., C1) were broadly repressed during infection, and hormone- and stress-responsive clusters (C2, C5, and C7) exhibited dynamic but inconsistent genotype differences. Collectively, while *V. dahliae* triggers widespread transcriptional reprogramming in both cultivars, the C8 module is distinguished by its persistent and coordinated activation in the Jinken1775, supporting its central role in mediating VW resistance.

**Figure 3:**
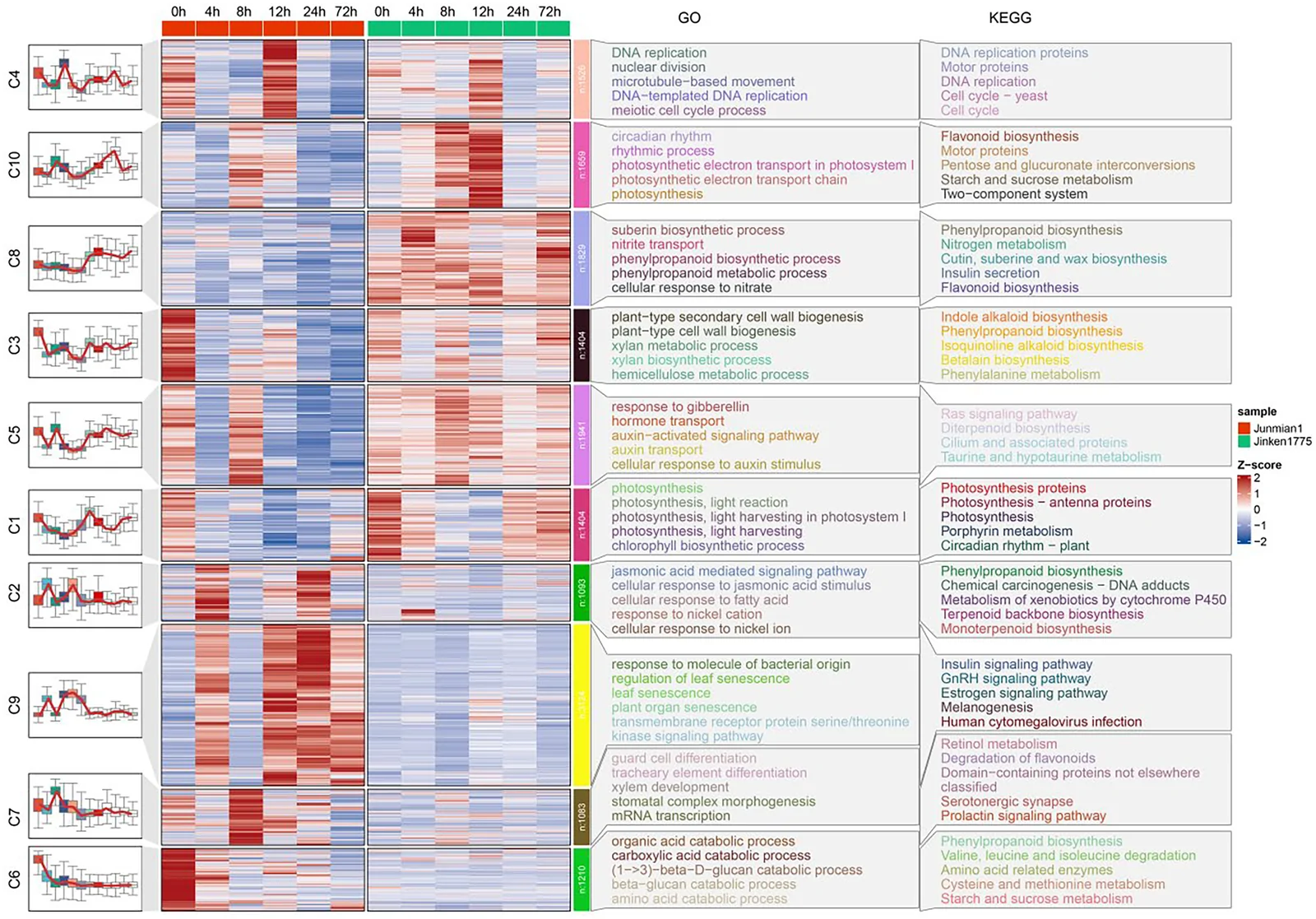
K-means clustering of DEGs in cotton after *V. dahliae* infection. The DEGs were grouped into ten clusters (C1–C10), with a heatmap illustrating their dynamic expression changes, along with GO and KEGG enrichment analyses that highlight representative genes and enriched biological process terms for each cluster.

### 3.3 Metabolomic reprogramming of Jinken1775 and Junmian1 in response to *V. dahliae* infection

To characterize metabolic responses to *V. dahliae* infection, untargeted metabolomic profiling identified 1, 498 DAMs (MSI Level 2) across all samples. These metabolites were classified into five major chemical categories: terpenoids; phenolic and aromatic compounds; nitrogen-containing compounds; lipids and fatty acid derivatives; and carbohydrates and their derivatives. Among them, flavonoids and terpenoids were the most abundant classes, followed by alkaloids and phenolic acids, highlighting the dominance of defense-related metabolites in cotton roots. The PCA showed clear separation among treatments and genotypes, with PC1 and PC2 explaining ~36% of the total variance (**Figure 2C**), indicating pronounced, time-dependent metabolic reprogramming after *V. dahliae* infection that was strongly shaped by genetic background. Consistently, OPLS-DA further resolved infected and control samples, supporting robust discrimination of metabolic states. Pairwise comparisons between Jinken1775 and Junmian1 at corresponding time points revealed 860, 845, 961, 937, 955, and 946 DAMs (**Figure 2D**). Notably, the number of up-regulated metabolites peaked at 12 hpi, suggesting a rapid metabolic activation phase during early infection. These results indicate that VW infection induces dynamic yet coordinated metabolic reprogramming in cotton, with the resistant genotype exhibiting a more focused and stable adjustment rather than indiscriminate metabolic disruption.

To further resolve temporal metabolic programs, all DAMs were subjected to K-means clustering based on accumulation trajectories. Seven subclasses were identified, revealing distinct temporal patterns and genotype-dependent divergence (**Figure 4**). Subclass C2 showed a pronounced transient induction at 12 hpi in Jinken1775, reflecting an early-to-mid stage metabolic activation. Subclass C5 displayed persistently higher metabolite abundances in Jinken1775 across multiple stages, defining a resistance-associated metabolic module. In contrast, subclasses C3 and C7 accumulated preferentially in Junmian1, whereas subclasses C1, C4, and C6 exhibited largely comparable patterns between the two cultivars, representing conserved metabolic responses to infection. Collectively, VW infection triggers both shared and genotype-specific metabolic reprogramming in cotton. The preferential and sustained activation of C2 and especially C5 in Jinken1775 highlights a resistance-biased metabolic signature, and metabolites within these subclasses were therefore prioritized for downstream integrative analyses to uncover key biochemical components underlying VW resistance.

**Figure 4:**
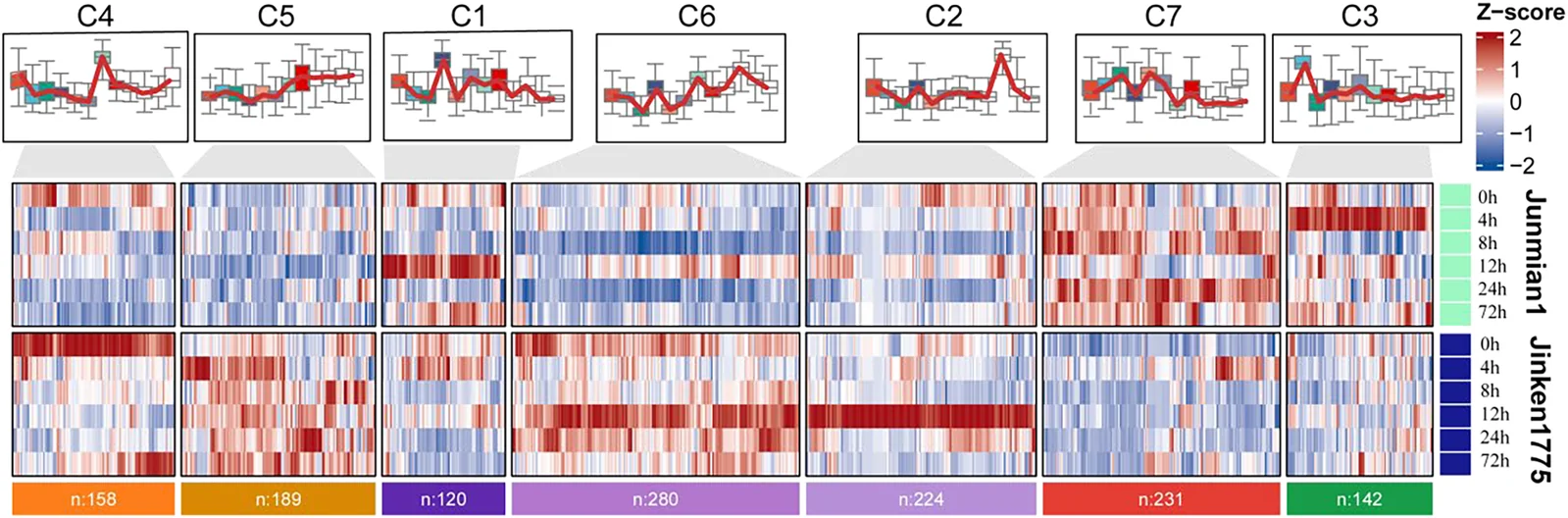
K-means clustering of DAMs in cotton after *V. dahliae* infection. The DAMs were grouped into seven clusters (C1–C7); the upper panels show mean metabolite abundance trends, and the lower heatmaps display Z-score normalized metabolite profiles.

### 3.4 Identification of key modules involved in cotton responses to *V. dahliae* infection

To dissect the coordination between transcriptional and metabolic regulation during VW infection, WGCNA was performed using the DEGs in Cluster 8, with metabolite abundances in Clusters 2 and 5 treated as external traits from Jinken1775 and Junmian1. Hierarchical clustering resolved 27 co-expression modules (**Figure 5A**). Module-metabolite correlation analysis identified three modules significantly associated with resistance-related metabolites (*r* > 0.85, FDR < 0.01) (**Figure 5B**). The MEblue module was strongly correlated with flavonoid compounds, including flavone, batatasin III, and peucenin. In contrast, the MElightyellow and MEcyan modules were mainly associated with lipid-derived metabolites, such as 12-methyltridecanoic acid, cis-9-palmitoleic acid, dodecanamide, and myristic acid. GO enrichment showed that MEblue genes were involved in water transport and cutin biosynthesis (**Figure 5C**), MElightyellow genes in stress responses (e.g., hypoxia response) (**Figure 5D**), and MEcyan genes in DNA replication and chromatin organization (**Figure 5E**) (**Supplementary Table S3**). Consistently, KEGG analysis revealed enrichment of diterpenoid biosynthesis in MEblue (**Supplementary Figure S2A**), fatty acid and lipid metabolism in MElightyellow (**Supplementary Figure S2B**), and necroptosis in MEcyan (**Supplementary Figure S2C**; **Supplementary Table S4**), suggesting activation of metabolic reprogramming and cell death-associated defense. Collectively, these results highlight flavonoid and lipid metabolism as major contributors to VW resistance in cotton.

**Figure 5:**
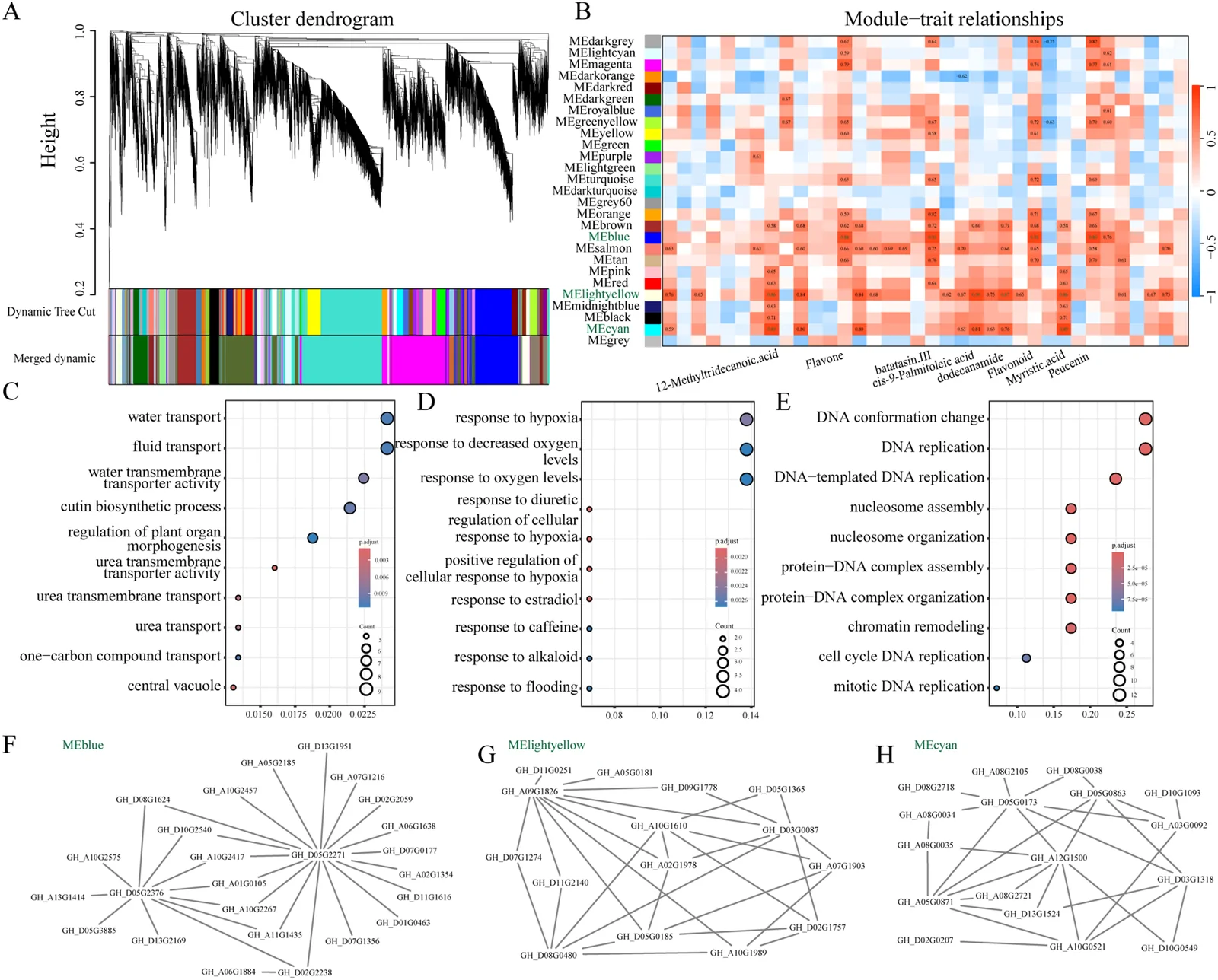
WGCNA-based association analysis between upregulated DEGs and DAMs in Junmian1 and Jinken1775. **(A)** Clustering dendrogram showing 27 modules of co-expressed genes. **(B)** Heatmap of correlations between modules and DAMs (color scale indicates correlation strength). **(C–E)** GO enrichment analysis of genes in three representative modules (MEblue, MElightcyan, and MEcyan) that showed strong correlations (*r* > 0.85) with eight key DAMs. **(F–H)** Co-expression networks of core genes in the MEblue, MElightcyan, and MEcyan module, respectively.

To further identify key regulatory genes, co-expression networks were constructed for each module. Genes with a module membership correlation coefficient above 0.85 were defined as hub genes. Using this threshold, 25 hub genes were detected in MEblue, including *WRKY33* (*GH_D07G0177*) and an F-box protein (*GH_D05G2271*) (**Figure 5F**). The MElightyellow module contained 15 hub genes, notably *CDPK28* (*GH_D03G0087*), Clp protease 5 (*GH_D07G1274*), and a lipid transfer protein (*GH_D11G0251*) (**Figure 5G**). In MEcyan, key hubs included *HMGB6* (*GH_A12G1500*) and histone superfamily members (*GH_D05G0173*, *GH_A05G0871*) (**Figure 5H**), implicating chromatin-associated regulation in VW defense.

### 3.5 Genes and metabolites involved in lipid and flavonoid metabolism

Integrated DEG-DAM analysis identified 14 hub candidate genes potentially associated with cotton resistance to *V. dahliae*, which were organized into a regulatory framework based on functional annotation (**Figures 6A–C**). In this model, pathogen perception is initiated by pattern-recognition receptors, such as the LRR-RLK, followed by activation of Ca²^+^-mediated signaling through CDPK28 and downstream transcriptional regulators, including WRKY33. These events drive transcriptional reprogramming during early defense. Genes involved in membrane remodeling and intracellular transport (e.g., *GH_A08G0034*, *GH_A08G0035*, and *GH_D02G2059*) likely contribute to vesicle trafficking, ion homeostasis, and cell barrier reinforcement. Meanwhile, regulators of protein homeostasis (e.g., *GH_A05G0181*, *GH_D07G127*4, and *GH_D13G1524*) may ensure proper synthesis and turnover of defense proteins. In parallel, several pathogen-responsive genes, including lipid transfer proteins and pathogenesis-related proteins (e.g., *GH_D11G0251* and *GH_A10G1989*), were identified as key downstream components of the defense network. Notably, several hub genes marked with asterisks have been previously validated as *V. dahliae* resistance genes in cotton, supporting the robustness of the integrative analysis. Overall, this transcriptome-metabolome framework reveals a coordinated defense program involving pathogen recognition, Ca²^+^ signaling, membrane and metabolic remodeling, and activation of antimicrobial responses, providing a mechanistic basis for lipid- and flavonoid-associated resistance pathways.

**Figure 6:**
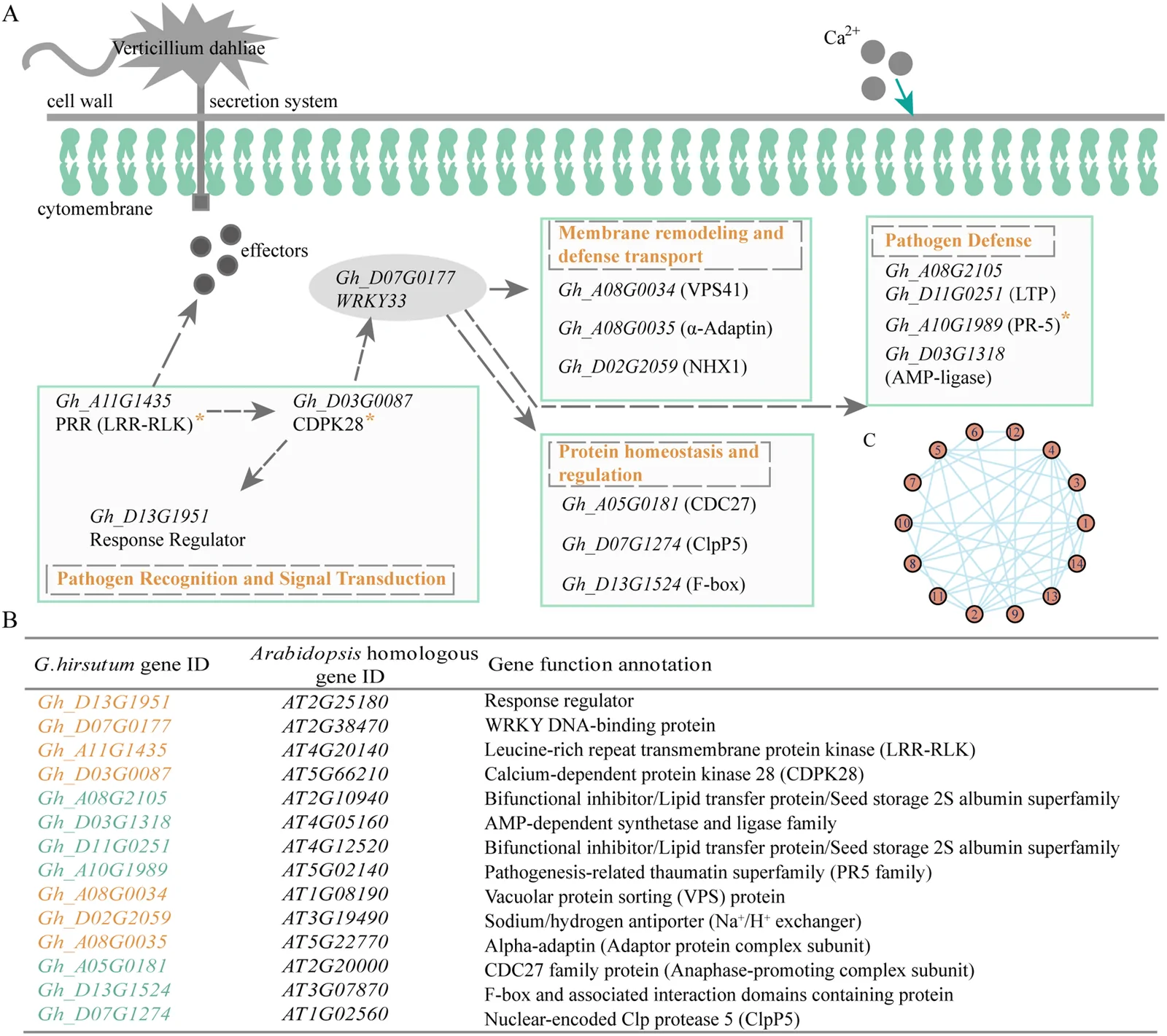
A proposed resistance model for VW in cotton. **(A)** Putative defense pathway. Pathogen perception by PRR activates Ca²^+^ signaling via CDPK28, which induces WRKY33-mediated transcription of downstream defenses. These include antimicrobial effectors, membrane remodeling/transport components, and protein homeostasis regulators. Genes marked with asterisks have been experimentally validated for VW resistance in cotton. **(B)** Summary of 14 VW-related candidate genes. **(C)** Co-expression network of the 14 candidate genes.

### 3.6 *GhWRKY33* positively regulates *V. dahliae* resistance in cotton

To validate key hub genes, RT-qPCR was performed on four representative candidates, including *GhWRKY33* (primers in **Supplementary Table S2**). Their expression profiles were highly consistent with RNA-seq data, confirming the reliability of the transcriptomic analysis (**Figures 7A–D**). To functionally characterize *GhWRKY33*, virus-induced gene silencing was conducted in the VW-resistant cultivar Jinken1775. Compared with the control, *TRV: GhWRKY33* plants exhibited pronounced chlorosis (**Figure 7E**). qRT-PCR analysis confirmed that *GhWRKY33* transcript level was significantly reduced, indicating efficient silencing (**Figure 7F**). Consistently, the disease index of *GhWRKY33*-silenced plants was significantly higher than that of *TRV:00* controls (**Figure 7G**). Moreover, longitudinal sections of the stems revealed substantially more severe vascular browning in *GhWRKY33*-silenced plants than in the controls (**Figure 7H**). Fungal recovery assays further showed markedly greater *V. dahliae* outgrowth from stem segments of *TRV: GhWRKY33* plants, indicating increased fungal colonization after *GhWRKY33* silencing (**Figure 7I**). Together, these results demonstrate that suppression of *GhWRKY33* compromises cotton resistance to *V. dahliae*, supporting its role as a positive regulator of VW defense.

**Figure 7:**
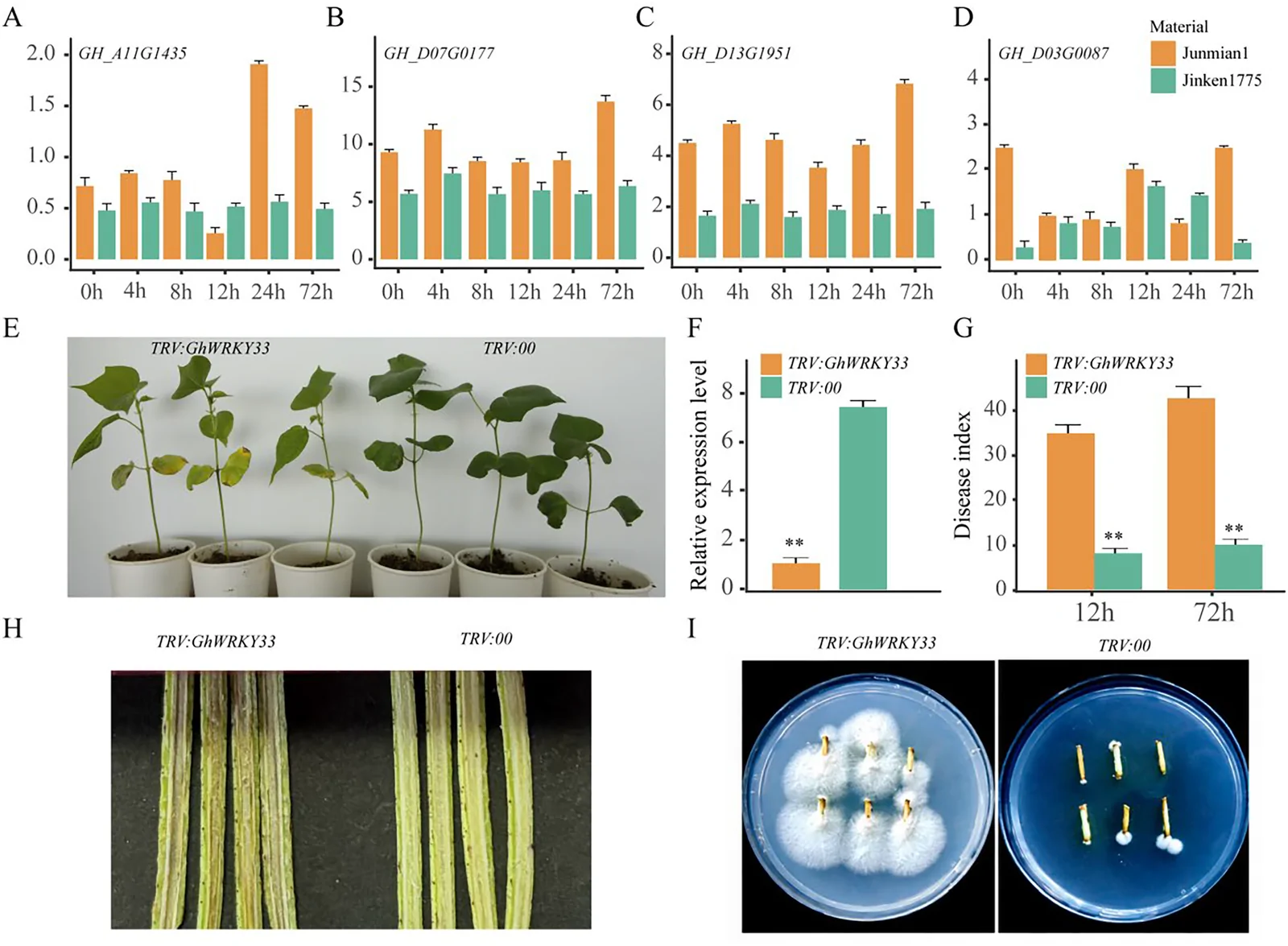
qRT-PCR and VIGS validation of key candidate genes involved in *V. dahliae* resistance. **(A–D)** Relative expression levels of four candidate genes in Junmian1 and Jinken1775 at 0, 4, 8, 12, 24, and 72 h post-inoculation (hpi) with *V. dahliae*, as determined by qRT-PCR. **(E)** Disease phenotypes of *GhWRKY33*-silenced (*TRV: GhWRKY33*) and empty-vector control (*TRV:00*) plants following *V. dahliae* inoculation. **(F)** Relative expression level of *GhWRKY33* in *TRV: GhWRKY33* and *TRV:00* plants. **(G)** Disease indices of *TRV: GhWRKY33* and *TRV:00* plants following *V. dahliae* inoculation. **(H)** Vascular browning in longitudinal stem sections of *TRV: GhWRKY33* and *TRV:00* plants. **(I)** Fungal recovery assay showing *V. dahliae* colonization in stem segments from *TRV: GhWRKY33* and *TRV:00* plants. ** indicates a significant difference at *P* < 0.01.

### 3.7 Natural variation and resistance potential of *GhWRKY33*

To assess natural variation and breeding potential of *GhWRKY33*, sequence polymorphisms were analyzed in a panel of 419 *Gossypium hirsutum* accessions. These accessions were evaluated for VW resistance at two locations, and classified as resistant (Group 2, mean disease index ≤ 35) or susceptible (Group 1, mean disease index > 35) based on the average disease index (DI) after *V. dahliae* inoculation. Haplotype analysis based on SNP variation across the *GhWRKY33* locus identified 28 haplotypes (**Figure 8A**), of which Hap1-Hap6 were predominant and accounted for most accessions in VW-susceptible (Group 1) and VW-resistant (Group 2) population (**Figures 8B, C**). Haplotype distribution differed between subgroups, with Group2 enriched for Hap1 and Hap2, whereas Group1 exhibited greater haplotype diversity (**Figure 8C**). Network analysis showed that major haplotypes were closely related, indicating limited but structured diversification of *GhWRKY33* during cotton improvement (**Figure 8D**). Importantly, the predominant haplotypes were preferentially associated with VW-resistant accessions, suggesting that favorable *GhWRKY33* alleles were more frequent in VW-resistant accessions, suggesting their potential association with resistance.

**Figure 8:**
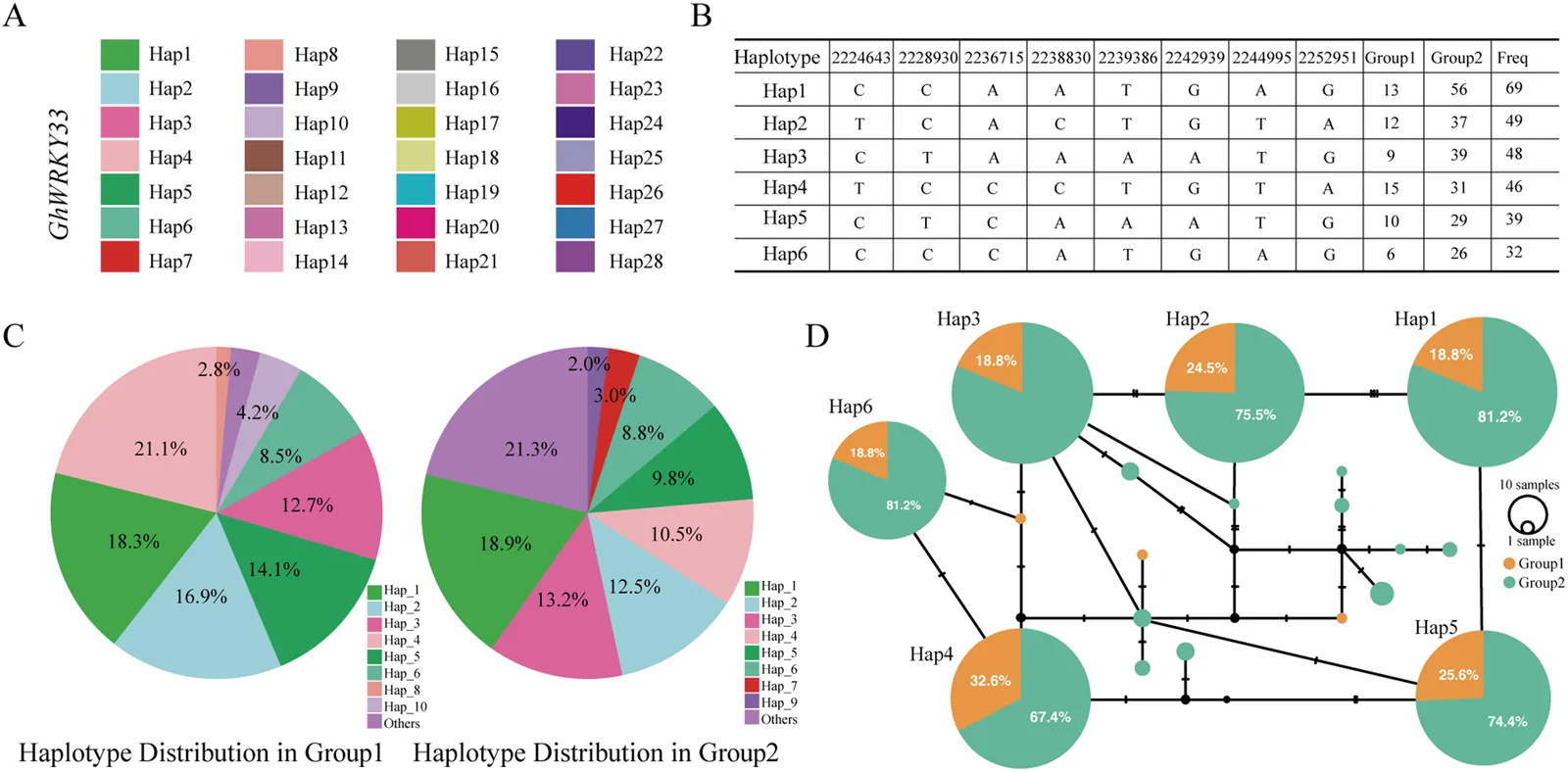
Haplotype combinations associated with cotton VW resistance. **(A)** Haplotype diversity and distribution of *GhWRKY33* in a panel of 419 cotton core germplasm. **(B)** Proportions of the 28 identified haplotypes (*GhWRKY33*^Hap1^-*GhWRKY33*^Hap28^) in VW-susceptible (Group 1) and VW-resistant (Group 2) cotton accessions. **(C)** SNP patterns defining the six major GhWRKY33 haplotypes and their frequencies in Group 1 and Group 2. **(D)** Haplotype network of *GhWRKY33*. Circle size is proportional to the number of accessions per haplotype.

## 4 Discussion

### 4.1 Early transcriptional and metabolic differences associated with *Verticillium wilt* resistance

Integration of transcriptomics and metabolomics provides a framework for investigating molecular changes during plant–pathogen interactions (Pan et al., 2024). Here, time-resolved profiling revealed distinct transcriptional and metabolic patterns in Jinken1775 and Junmian1 during the early stages following *V. dahliae* inoculation. Differences already evident at 0 h indicate that these patterns reflect both constitutive variation between the cultivars and subsequent responses to inoculation. Against these different basal backgrounds, Junmian1 exhibited more extensive transcriptional changes, whereas Jinken1775 showed a comparatively limited response. This contrast suggests that the breadth of transcriptional reprogramming alone does not explain resistance and highlights the potential importance of response timing and functional composition (Zhang et al., 2023). The extensive changes in Junmian1 may represent compensatory responses that fail to restrict infection effectively or arise from greater pathogen pressure and associated cellular damage. Such responses could involve substantial resource reallocation and disturbance of cellular homeostasis as disease progresses (Wang et al., 2021; Xiong et al., 2021). Conversely, the more limited changes in Jinken1775 may reflect differences in basal defense capacity or reduced physiological disturbance following inoculation. Although DEG counts cannot establish regulatory precision or energetic efficiency, these alternative interpretations provide a basis for examining how early response dynamics relate to subsequent disease development. DEGs between one sample were identified by comparing each post-inoculation time point with the untreated 0-h sample of the same cultivar rather than against mock controls, since mock inoculation of this vascular pathogen itself induces non-specific stress and the 0-h baseline ensured a consistent criterion across time points. We acknowledge that the absence of parallel mock controls does not fully exclude time-dependent effects, which parallel mock-inoculated experiments could help resolve.

The integrated analyses further linked transcriptional differences to metabolic variation involving calcium signaling, membrane-related processes and secondary metabolism. In Jinken1775, resistance-associated expression patterns, including those represented by the C8 cluster, were accompanied by differences in the abundance of metabolites annotated as flavonoids and lipid derivatives. The established roles of these compound classes in antimicrobial defense, ROS buffering, membrane function and defense signaling provide biological context for their potential involvement in resistance (Xiong et al., 2021; Zhang et al., 2023). Moreover, hundreds of DAMs exhibited genotype- and time-dependent variation, adding a metabolic dimension to the transcriptional responses and supporting further investigation of metabolic reprogramming in cotton immunity (La Camera et al., 2004). Together, these observations suggest that contrasting resistance phenotypes may involve interactions between basal molecular states and subsequent signaling and metabolic adjustments. The inferred gene–metabolite associations identify candidate components of these processes for functional testing, while their causal relationships remain to be established.

### 4.2 *GhWRKY33*-associated defense networks and breeding implications

Metabolic reprogramming is a major component of plant immunity, with flavonoids and lipid-derived compounds contributing to antimicrobial defense, redox homeostasis, membrane remodeling, and defense signaling (Luo et al., 2021; Xiong et al., 2021). In the present study, Jinken1775 exhibited preferential and sustained accumulation of several flavonoid- and lipid-related metabolites compared with Junmian1. Consistently, metabolite clustering and WGCNA identified resistance-associated modules correlated with flavonoid and lipid-derived compounds and enriched in stress-responsive and membrane-associated processes. Within these networks, *GhWRKY33* was prioritized as a candidate hub associated with resistance-related metabolic variation. Silencing *GhWRKY33* in Jinken1775 increased disease severity, vascular browning, and fungal colonization following V. dahliae inoculation, supporting a positive contribution of *GhWRKY33* to Verticillium wilt resistance under the conditions tested. This result is consistent with previous cotton VIGS-based evidence (Zhang et al., 2016). By contrast, heterologous overexpression of *GhWRKY33* in *Arabidopsis thaliana* was reported to increase susceptibility to *V. dahliae* (Ji et al., 2023). Differences in host background, gene dosage, or experimental conditions may contribute to these contrasting observations. Importantly, the present co-expression and metabolite associations do not demonstrate direct transcriptional regulation by *GhWRKY33*, nor do the VIGS data establish that lipid or flavonoid changes directly mediate the resistance phenotype. Thus, the network should be viewed as a hypothesis-generating framework rather than a fully resolved regulatory pathway.

Several limitations should be considered when interpreting the function of *GhWRKY33*. Its contribution to resistance was evaluated only in the resistant cultivar Jinken1775 and against a single *V. dahliae* isolate, V991. Therefore, whether the observed effect is conserved across cotton genetic backgrounds, pathogen isolates, defoliating and non-defoliating pathotypes, or distinct races cannot be determined from the present study. Evaluation of *GhWRKY33* perturbation and contrasting haplotypes across multiple cotton backgrounds and independently characterized V. dahliae isolates will be necessary to assess the stability and breadth of the resistance effect and to identify potential genotype-by-isolate interactions. In addition, transcriptomic and metabolomic profiling of *GhWRKY33*-silenced plants, together with promoter-binding, ChIP-based, and transactivation assays, will be required to identify direct downstream targets and determine whether the lipid- and flavonoid-associated changes inferred from the current network are mechanistically linked to resistance.

The natural-variation analysis further extends the functional observations toward potential breeding application. *GhWRKY33* haplotypes showed different distributions between resistant and susceptible accessions, suggesting that sequence variation at this locus may contribute to phenotypic variation in Verticillium wilt resistance. However, these associations should first be validated in independent germplasm panels and segregating populations while accounting for population structure and relatedness. Informative SNPs or indels distinguishing resistance-associated haplotypes could subsequently be converted into locus-specific markers, such as kompetitive allele-specific PCR (KASP) assays. Following validation, such markers could facilitate the identification of donor parents, marker-assisted selection of resistant progeny, and pyramiding of *GhWRKY33*-associated alleles with other validated Verticillium wilt resistance loci. Evaluation across pathogen isolates, genetic backgrounds, and environments, together with assessment of yield and fiber-quality effects, will ultimately determine their practical value in cotton breeding.

The haplotype findings provide a starting point for translating *GhWRKY33* research into marker-assisted selection (MAS). The observed relationships between haplotype distribution and resistance should first be validated in independent germplasm panels, accounting for population structure and relatedness, and in segregating populations. Informative SNPs or indels could then be converted into locus-specific markers, such as kompetitive allele-specific PCR (KASP) assays, with their ability to distinguish the target haplotypes verified against sequence data. Following validation, these markers could support the identification of donor parents and selection of progeny carrying resistance-associated haplotypes during crossing or backcrossing. They could also facilitate combining this locus with other validated resistance loci. Evaluation across pathogen isolates, genetic backgrounds and environments, together with assessments of yield and fiber quality, would establish their practical breeding value. Thus, the current findings nominate *GhWRKY33* haplotypes for marker development and validation.

## Data Availability

The datasets presented in this study can be found in online repositories. The names of the repository/repositories and accession number(s) can be found below: https://www.ncbi.nlm.nih.gov/, PRJNA1522644.

## Supplementary material

Supplementary Figure 1 UpSet plots of DEGs between Jinken1775 and Junmian1 at different time points after *V. dahliae* infection. **(A)** UpSet plot of up-regulated DEGs. **(B)** UpSet plot of down-regulated DEGs.

Supplementary Figure 2 KEGG enrichment analysis of module-specific genes from WGCNA in cotton response to *V. dahliae* infection. **(A)** KEGG enrichment results for the MEblue module, associated with flavonoids and phenolic compounds. Key enriched pathways include diterpenoid biosynthesis and sulfur metabolism. **(B)** KEGG enrichment results for the MElightcyan module, linked to lipid compounds. Prominent pathways involve glycolysis/gluconeogenesis, pentose and glucuronate interconversions, biosynthesis of unsaturated fatty acids, and fatty acids biosynthesis. **(C)** KEGG enrichment results for the MEcyan module. Enriched pathways include neutrophil extracellular trap formation, systemic lupus erythematosus, alcoholism, and DNA replication.

Supplementary Figure 3 RNA-seq expression profiles of 14 candidate genes in Junmian1 and Jinken1775 at different time points after *V. dahliae* infection.
